# Stem traits promote wheat climate-resilience

**DOI:** 10.3389/fpls.2024.1388881

**Published:** 2024-07-25

**Authors:** Simeon Ntawuguranayo, Michael Zilberberg, Kamal Nashef, David J. Bonfil, Naresh Kumar Bainsla, Francisco J. Piñera-Chavez, Matthew Paul Reynolds, Zvi Peleg, Roi Ben-David

**Affiliations:** ^1^ The Robert H. Smith Faculty of Agriculture, Food and Environment, The Hebrew University of Jerusalem, Rehovot, Israel; ^2^ The Institute of Plant Sciences, Agriculture Research Organization (ARO) - Volcani Institute, Rishon LeZion, Israel; ^3^ The institute of plant sciences, Agricultural Research Organization, Gilat Research Center, Gilat, Israel; ^4^ Division of Genetics, Indian Agricultural Research Institute (IARI), New Delhi, India; ^5^ International Maize and Wheat Improvement Center (CIMMYT), Texcoco, Mexico

**Keywords:** grain filling, peduncle length, stem diameter, stem solidness, water-soluble carbohydrates, wheat breeding

## Abstract

**Introduction:**

Wheat grain filling processes under post-anthesis stress scenarios depend mainly on stem traits and remobilization of stem water-soluble carbohydrates (WSC).

**Methods:**

A diverse panel of advanced semi-dwarf spring wheat lines, representing a natural variation in stem traits (WSC content, stem diameter, peduncle length, and stem wall width), was used to identify specific traits that reliably reflect the relationship between WSC and grain yield. The panel was phenotyped under various environmental conditions: well-watered, water-limited, and heat stress in Mexico, and terminal-drought in Israel.

**Results:**

Environmental stresses reduced grain yield (from 626 g m^−2^ under well-watered to 213 g m^−2^ under heat), lower internode diameter, and peduncle length. However, stem-WSC generally peaked 3-4 weeks after heading under all environmental conditions except heat (where it peaked earlier) and expressed the highest values under water-limited and terminal-drought environments. Increased investment in internode diameter and peduncle length was associated with a higher accumulation of stem WSC, which showed a positive association with yield and kernel weight. Across all environments, there were no apparent trade-offs between increased crop investment in internode diameter, peduncle length, and grain yield.

**Discussion:**

Our results showed that selecting for genotypes with higher resource investment in stem structural biomass, WSC accumulation, and remobilization could be a valuable strategy to ameliorate grain size reduction under stress without compromising grain yield potential. Furthermore, easy-to-measure proxies for WSC (stem diameter at specific internodes and length of the last internode, i.e., the peduncle) could significantly increase throughput, potentially at the breeding scale.

## 1 Introduction

Wheat (*Triticum* sp.) is a major staple food crop in both the Global North and South and the most widely grown crop at 700m ha worldwide (Braun et al., 2010). Water scarcity and heat stress are the primary environmental constraints affecting wheat growth and production and are increasingly exacerbated due to climatic fluctuations, which jeopardize future food security. Thus, to meet the global demand of the rising population, there is an urgent need to develop wheat cultivars that are better adapted to the changing environment. Thus, identifying novel adaptative traits and their underlying mechanisms will serve as promising genetic resources for breeding.

In cereals, the grain-filling rate depends on the photo-assimilates supply and the remobilization of pre-anthesis water-soluble carbohydrate (WSC) reserve content from the stem. Under optimal conditions, pre-anthesis assimilate reserves in the stem contribute up to 10**–**40% of the final grain weight (Dreccer et al., 2009), whereas, under stressful conditions, it can reach up to 40**–**70% (Gaur et al., 2022). Stem reserves from pre-anthesis assimilation, mainly in the form of fructan (additional forms are sucrose, glucose, and starch), have been recognized as an essential source of carbon for grain filling when current photosynthesis is inhibited by abiotic stress (Fischer, 2011). Moreover, the efficiency of reserve mobilization, especially under terminal-drought, might also impact the grain yield (Morgun et al., 2022).

Stem reserve mobilization is a dynamic process, and while it serves as a strong sink at pre-anthesis, during the grain-filling phase, it turns into a source for the later remobilization process (Blum, 1998). Under water stress conditions, the mobilization of the reserves is controlled by stem fructan exohydrolase activity (FEH), which hydrolyses the stored fructans into mobile fructose (Wardlaw and Willenbrink, 2000; Zhang et al., 2008). Wheat genotypes with higher stem WSC reserves and mobilization rate were more adapted to grain filling under heat stress (Talukder et al., 2013) and water stress (Yáñez et al., 2017). A wide genotypic variation at the onset of grain growth was found for WSC, and a complex genetic control with a moderate to high narrow-sense heritability suggests that phenotypic selection will be the preferred approach to improve this trait (Fischer, 2011). Studies on water stress during grain filling indicate that higher WSC storage capacity does not always indicate higher grain-filling, as the remobilization rate differs among genotypes of the same stem reserve storage capacity (Zhang et al., 2009). Therefore, Blum (2011) suggests an independent genotype selection for WSC storage capacity (selecting for high WSC content) and WSC mobilization (selecting for grain size under desiccation treatment).

In the last decade, spring wheat breeders have optimized biomass partitioning close to its upper limit with values between ~0.50 and ~0.62 for actual and hypothetical harvest index (HI) (Bainsla et al., 2020). The current urgent challenge is to maximize biomass production while maintaining optimal biomass partitioning. Canopy traits could include sink-source dynamics during stem elongation and spike development. It is unclear how stem morphology (stem width, stem wall width, peduncle length, etc.) might be associated with stem source strength and its role in defining sink (i.e., ear) development. The solid stem was associated with enhanced source capacity via higher WSC, which provides better resilience to abiotic stress during grain filling (Saint Pierre et al., 2010). Moreover, Ahmed et al. (2014) found a strong association between stem diameter with thousand kernel weight, single grain weight, and grain yield per spike under drought stress, indicating the importance of this trait in sustaining gain filling through the provision of greater stem capacity for storing assimilates before mobilizing it to grains.

The stem internode length and weight also affect stem reserve accumulation and remobilization, suggesting that larger stem diameter and stem density would be advantageous for grain filling under stress conditions (Ehdaie et al., 2006b). The peduncle (i.e., the uppermost internode) is an important organ for WSC storage, as the largest pool of carbon and nitrogen during grain filling (Martínez−Peña et al., 2023). The peduncle length is closely associated with inflorescence development and reserve transportation into the spike and grains (Reynolds et al., 2009). The peduncle dry weight is positively associated with peduncle WSC content (Talukder et al., 2013), and higher peduncle WSC content can contribute to better adaptability to heat stress.

Our working hypothesis was that genotypes with high levels of stem WSC around anthesis and high WSC mobilization to the developing grains are associated with heat and water stress adaptability during the critical grain-filling stage. To identify stem traits that reliably reflect the relationship between WSC and grain yield, we evaluated a diverse set of spring wheat, developed for natural variations in stem morphology and stem WSC accumulation at anthesis and post-anthesis, under various environmental conditions. The current study aimed to: (*i*) Test the association between stem characteristics and WSC reserves. (*ii*) Characterize the dynamic of WSC in the stem during grain filling from anthesis to maturity. (*iii*) Test the association between stem traits and yield components under different environmental conditions.

## 2 Materials and methods

### 2.1 Plant material

The panel used in this study includes 25 breeding lines of spring wheat selected from the International Maize and Wheat Improvement Centre (CIMMYT) collection based on differences in stem solidness, width, and water-soluble carbohydrates (WSC) (**Supplementary Table S1**). To prevent any confounding effects, emphasis was given to lines with minimum variability for phenology and plant height traits.

### 2.2 Experimental design and environmental conditions

Four field experiments were conducted. Three environments at Campo Experimental Norman E. Borlaug (CENEB) in the Valle del Yaqui, Obregon, Mexico (27^◦^24^◦^N; 109^◦^56^◦^W; 38 m above sea level) during the 2018–2019 growing season: Well-watered (WW, with a total of 526.4 mm where 100 mm was applied before sowing, 26.4 mm of seasonal rainfall, and 400 mm applied as supplemental flood irrigation for entire growing season), water-limited (WL, with a total of 166.3 mm, where 100 mm applied before sowing, 26.3 mm seasonal rainfall, and 40 mm applied as supplemental flood irrigation), and heat where the crops were sown at the beginning of March so that they can grow under high temperature throughout (with total irrigation of 506.7 mm including pre-sowing irrigation of 100 mm, 6.7 mm rainfall and 400 mm supplemental flood irrigation applied during the growing season). The site is temperate, and the soil is a montmorillonitic typic Caliciorthid (coarse sandy clay), low in organic matter and slightly alkaline (pH 7.7). The fourth environment was a rain-fed Mediterranean, referred to as terminal-drought (TD). This environment is characterized by low soil moisture and heat waves during the grain-filling stage, with a total precipitation of 564 mm during the winter of 2020–21 season (Net house, Rishon LeZion, Israel). The seasonal temperatures, precipitation, and supplemental irrigations are presented in **Supplementary Figure S1**. The experiments were sown on 28 November-2018 (WW) and 03-December- 2018 (WL), 05-March-2019 (Heat), and on 11- November-2020 (TD).

α-lattice design (Falconer, 1989) with three replicates was used for WW, WL, and heat environments (*n*=3). For the TD environment, a randomized block design was employed (*n*=3). Plants were sown in 2-row beds plot of 2 m, the distance between rows within a plot was 0.25 m, and the distance between adjacent plots was 0.5 m across all experiments. A seeding rate of 220 plants m^-2^ was employed. All plots received single fertilization of 50 kg N ha^–1^ (Urea) and 40 kg P ha^–1^ (triple superphosphate) before sowing in all environments.

The fields were treated with fungicides and pesticides periodically to prevent the development of fungal pathogens or insect pests and were manually weeded monthly. The growing season from sowing to harvesting lasted 160 days for WW, 145 days for WL, 127 days for Heat, and 210 days for TD.

### 2.3 Phenotyping stem traits

#### 2.3.1 Measuring stem WSC

At 15 and 25 days after heading (DAH) and at maturity, five primary main stems were selected randomly from each plot, avoiding plot edges. Stem samples were separated from spikes and oven-dried at 65°C for 48 hours. After weighing the total plant dry matter (DM), leaf sheaths were carefully removed from the stems and weighed again to record stem DM. Stem-dried samples were ground using a sieve mesh of 0.5 mm. Samples were then scanned for WSC content using Near-Infrared Spectroscopy (NIRSystems 6500 FOSS), and the spectral signature was calculated. The software WINISI FOSS was used to estimate the concentration of soluble sugars. Stem WSC was calibrated separately as a ratio per growing degree days (GDD) in each environment. From the data of the WSC on each of the measurement dates, it was possible to calculate the change in concentration over time in each line and to examine the effect on the weight of the final grains. WSC accumulation = WSC at the peak, and WSC remobilization was obtained by WSC at the peak minus WSC at maturity.

#### 2.3.2 Measuring stem characteristics

**Figure 1:**
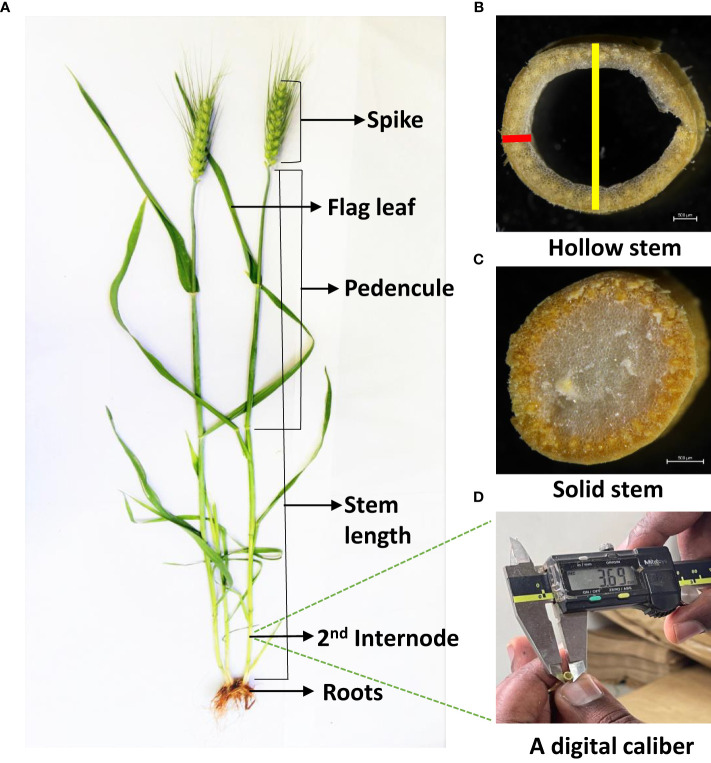
Display of measured wheat stem traits. **(A)** Wheat main parts, **(B)** Hollow stem at second internode; **(C)** Solid stem at second internode; **(D)** Digital caliber used to measure diameter and wall width. The yellow line shows the measured stem diameter, and the red line shows the measured wall width.


When sampling for stem DM and WSC at 25 DAH, five main stems with leaf sheath removed were used for recording internode diameter (ID), internode wall width (IWW), stem solidness score (0 for complete hollow and 1 for complete solid stem) and peduncle length (PL-the uppermost internode). Following the methodology outlined by Piñera−Chavez et al. (2016) on measuring stem characters, ID and IWW were measured in the middle of the second internode using digital calipers (**Figure 1**). From the measurement data, stem solidness was found by the following formula:

Stem solidness=Stem area−Hollow area

$$Stem\;area=3.14\times {\left(\frac{Stem\;diameter}{2}\right)}^{2}$$

$$Hollow\;area=3.14\times {\left(\frac{Hollow\;diameter}{2}\right)}^{2}$$

Hollow diameter=Stem diameter−(Stem wall thickness×2)

#### 2.3.3 Additional crop trait characterization and yield components assessment

Plants were characterized for phenology traits: Days to heading (DTH) were calculated from emergence to heading and were converted to Growing Degree Days (GDD) for each environment separately. The heading date was recorded when 80% of the spikes in the plot were fully exposed above the flag leaf, Zadoks stage 59 (Zadoks et al., 1974). Plant height (PH) was measured before harvest using a ruler from the base of the plant to the terminal spikelet.

Yield components: Grain yield (GY), grain number (GN), and thousand-grain weight (TGW) were counted manually and weighted using a digital scale. Dry biomass (BM) at physiological maturity calculated with yield components was recorded based on random samples of 0.25 m^2^ in each plot (*n*=3) and was calculated as gram per m^2^. The harvest index (HI) was calculated from a manual harvest of 0.25 m^2^ (weight ratio of the grains to total above-ground biomass). Each plot was manually harvested for grain yield.

### 2.4 Data analysis

The JMP^®^ ver. 17.0 pro statistical package (SAS Institute, Cary, NC, USA) was used for all statistical analyses. Descriptive statistics were performed on the full dataset to illustrate the variable distribution and to calculate the coefficient of variance (CV). Analysis of Variance (ANOVA) was used to assess the possible effect of genotype (G), environment (E), and G×E interactions on the performance of the genotype. Principle Component Analysis (PCA) was performed based on a correlation matrix. Initially, highly auto-correlated variables (when the vector was overlapping) were removed from the analysis. Broad-sense heritability (*_bs_h* ^2^) was estimated using ANOVA-based variance components *_bs_h* ^2^ = V_G_ **/**(V_G_ + V_GE_ + V_e_), where V_G_ = Genotypic variance, V_GE_ = genotype by environment variance, and V_e_ is the residual.

## 3 Results

### 3.1 Stem traits are affected by complex genotype *×* environment interactions

**Figure 2:**
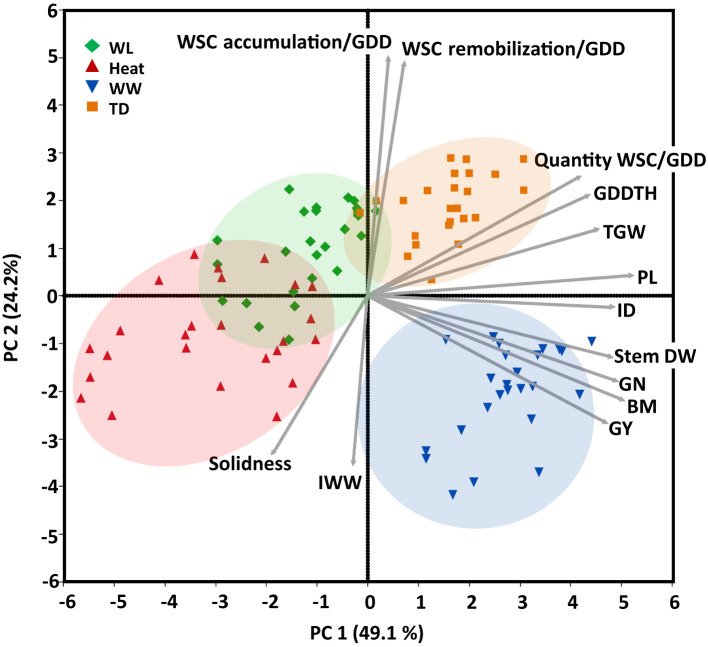
Principal components analysis (based on correlation matrix) of continuous phenotypic and yield components recorded for 25 genotypes under four environments: water-limited (WL, green); Heat stress (Heat, red), well-watered (WW, blue), and terminal-drought (TD, orange). Biplot vectors are trait factor loadings for principal component (PC) 1 and PC2. Growing degree days to heading (GDDTH), thousand-grain weight (TGW), grain number GN), quantity of water-soluble carbohydrates per growing degree days (Quantity WSC per shoot/GDD), water-soluble carbohydrate accumulation and remobilization per shoot/GDD, peduncle length (PL), individual stem dry weight at the peak of water-soluble carbohydrates (Stem DW, g), internode diameter (ID), biomass (BM), grain yield (GY) and internode wall width (IWW).

PCA of agronomic, stem, and yield-related traits extracted two principal components (Eigenvalues >1), accounting collectively for 73.3% of phenotypic variance across all environments (**Figure 2**). PC1 (X-axis) explained 49.1% of the variation and was positively loaded with GY, BM, GN, stem DW, ID, PL, TGW, GDDTH, the quantity of WSC/GDD, WSC remobilization and accumulation/GDD and was negatively loaded with stem solidness. PC2 (Y-axis) explained 24.2% of the variation and was positively loaded with WSC remobilization and accumulation/GDD, quantity of WSC/GDD, GDDTH, TGW and negatively loaded with GY, BM, stem DW, ID, GN, and IWW. The amount of available water mainly drove the environments separation. The well-watered environment is clustered on the fourth quadrant of the PCA toward GY and BM. The water-limited (assembled in the second quadrant close to the third quadrant) and terminal-drought (clustered in the first quadrant) positively affected the accumulation and remobilization of water-soluble carbohydrates with a positive loading of both WSC accumulation, remobilization, and the quantity of water-soluble carbohydrates per growing degree days.

The heat (clustered close to the third quadrant) and water-limited environments negatively affected the stem traits (internode diameter and peduncle), yield traits (grain yield and thousand kernel weight), and biomass. Stem solidness and internode wall width negatively loaded in the third quadrant were positively affected by water-limited and heat stress.

The panel showed wide variability for stem characters under all four environments; IWW mean was 0.86, 0.75, and 0.61 mm for WW, WL, and heat environments, respectively, while in the TD environment, IWW was 0.48mm. Internode diameter showed a similar pattern with mean values of 4.22, 3.31, 3.19, and 3.98 mm for WW, WL, heat, and TD environments, respectively (**Table 1**). In addition, environments and genotypes significantly interact [*P*(F)< 0.0001] in both traits (**Table 2**). In the IWW trait, some genotypes showed stable performance across three environments except the TDS (e.g., lines C-SS-8, C-SS-16, and C-SS-23) (**Supplementary Table S2**). The environment highly affected the peduncle length, from the mean value of 39.5, and 39 cm under TD, WW to 27.4, 20.9, - cm under WL, and heat- respectively.

**Table 1:** Summary of the mean performance, standard error (SE), and coefficient of variance (CV) for the phenotypic traits of 25 genotypes under four environmental conditions: Well-watered, water-limited, heat stress, and terminal-drought.

Trait*^a^*	Well-watered		Water-limited		Heat stress		Terminal-drought	
	Mean ± SE	CV	Mean ± SE	CV	Mean ± SE	CV	Mean ± SE	CV
**GDDTH** (°C)	1262 ± 5.69	3.90	1133 ± 6.79	5.18	1022 ± 6.67	5.64	1400 ± 13.52	8.36
**Plant height (cm)**	101 ± 0.78	6.73	71.00 ± 0.73	8.99	62.00 ± 1.27	17.88	98.00 ± 0.84	7.46
**GDDTM (**°C)	2175 ± 5.47	2.17	1936 ± 6.97	3.11	1668 ± 8.17	4.24	2242 ± 10.96	4.23
**Peduncle length (cm)**	39.04 ± 0.37	8.29	27.44 ± 0.30	9.60	20.96 ± 0.47	19.72	39.56 ± 0.45	9.85
**Internode diameter (mm)**	4.22 ± 0.03	7.99	3.31 ± 0.03	10.15	3.18 ± 0.03	9.45	3.98 ± 0.07	15.55
**Internode wall width (mm)**	0.86 ± 0.04	43.10	0.75 ± 0.04	47.04	0.61 ± 0.02	40.81	0.48 ± 0.01	20.39
**Stem solidness (%)**	62.29 ± 1.9	26.54	65.60 ± 2.14	28.31	59.69 ± 0.02	29.48	43.31 ± 1.22	23.25
**Stem DW (g)**	2.89 ± 0.05	16.63	1.82 ± 0.03	15.35	1.35 ± 0.03	25.13	2.04 ± 0.06	27.48
**WSC accumulation per shoot/GDD (mg/°C)**	0.014 ± 0.01	15.05	0.02 ± 0.01	11.18	0.01 ± 0.01	21.72	0.03 ± 0.01	12.04
**WSC remobilization per shoot/GDD (mg/°C)**	0.011 ± 0.01	18.87	0.02 ± 0.01	10.91	0.01 ± 0.01	27.43	0.02 ± 0.01	13.45
**Harvest index**	0.44 ± 0.01	9.10	0.49 ± 0.01	9.06	0.39 ± 0.01	14.33	0.34 ± 0.35	11.85
**Biomass(g/m^2^)**	1413.4 ± 24.30	14.94	591.36 ± 17.02	24.92	522.40 ± 31.37	52.00	885.44 ± 4.49	35.16
**Grain yield ((g/m^2^)**	626.55 ± 8.01	11.07	287.02 ± 7.67	23.01	213.50 ± 13.85	56.19	311.60 ± 14.82	41.20
**Thousand-grain weight (g)**	46.6 ± 8.01	11.06	40.72 ± 0.49	10.59	35.72 ± 0.56	13.71	49.08 ± 0.68	12.14

^a^The phenotypic traits: Growing degree days to heading (GDDTH), growing degree days to maturity (GDDTM), individual stem dry weight at the peak of water-soluble carbohydrates (stem DW), water-soluble carbohydrates accumulation per growing degree days (WSC accumulation per shoot/GDD), and water-soluble carbohydrates remobilization per growing degree days (WSC remobilization per shoot/GDD).

**Table 2:** Analysis of variance for the effects of genotype (G), environments (E), and their interactions (G × E) on 25 genotypes under four environmental conditions: well-watered, water-limited, heat stress, and terminal-drought.

Trait*^a^*	Source	d.f.*^b^*	MS*^c^*	F Ratio	*Prob > F*	R square	*_bs_h* ^2^ *^d^*
GDDTH (^o^C)	Genotype	24	30121	74.1731	<.0001	0.99	0.25
	Environment	3	1990881	4902.488	<.0001		
	Genotype*Environment	72	12417	30.5758	<.0001		
Plant height (cm)	Genotype	24	330.98	15.4791	<.0001	0.96	0.29
	Environment	3	28929.31	1352.959	<.0001		
	Genotype*Environment	72	99.05	4.6321	<.0001		
GDDTM (^o^C)	Genotype	24	23052	8.8184	<.0001	0.97	0.29
	Environment	3	5094375	1948.824	<.0001		
	Genotype*Environment	72	5516	2.1101	<.0001		
Pedencule length (cm)	Genotype	24	67.099	14.251	<.0001	0.96	0.34
	Environment	3	6228.241	1322.793	<.0001		
	Genotype*Environment	72	15.688	3.3319	<.0001		
Internode diameter (mm)	Genotype	24	0.53399	6.3353	<.0001	0.85	0.13
	Environment	3	18.69812	221.8394	<.0001		
	Genotype*Environment	72	0.27454	3.2572	<.0001		
Stem Solidness (%)	Genotype	24	1541.144	17.1393	<.0001	0.82	0.4
	Environment	3	6674.832	74.2318	<.0001		
	Genotype*Environment	72	287.468	3.197	<.0001		
Internode wall width (mm)	Genotype	24	0.489888	17.4374	<.0001	0.82	0.38
	Environment	3	1.944447	69.2118	<.0001		
	Genotype*Environment	72	0.103703	3.6913	<.0001		
Stem DW (g)	Genotype	24	0.59258	7.3241	<.0001	0.89	0.13
	Environment	3	30.72106	379.7003	<.0001		
	Genotype*Environment	72	0.32165	3.9755	<.0001		
WSC Accumulation per shoot/GDD (mg/^o^C)	Genotype	24	0.0000224	6.1742	<.0001	0.88	0.13
	Environment	3	0.0012874	355.6418	<.0001		
	Genotype*Environment	72	0.0000109	3.02	<.0001		
WSC Remobilization per shoot/GDD (mg/^o^C)	Genotype	24	0.0000195	4.4631	<.0001	0.87	0.09
	Environment	3	0.0014755	338.3954	<.0001		
	Genotype*Environment	72	0.0000116	2.6566	<.0001		
Harvest index	Genotype	24	0.005532	5.5918	<.0001	0.87	0.05
	Environment	3	0.3078202	311.1493	<.0001		
	Genotype*Environment	72	0.0041157	4.1602	<.0001		
Biomass (g/m^2^)	Genotype	24	201174	5.7098	<.0001	0.87	0.17
	Environment	3	12322160	349.7319	<.0001		
	Genotype*Environment	72	78795	2.2364	<.0001		
Grain yield (g/m^2^)	Genotype	24	37899	6.6865	<.0001	0.89	0.21
	Environment	3	2504437	441.8561	<.0001		
	Genotype*Environment	72	12800	2.2583	<.0001		
Thousand-grain weight (g)	Genotype	24	228.303	58.6595	<.0001	0.95	0.64
	Environment	3	2701.209	694.0414	<.0001		
	Genotype*Environment	72	20.796	5.3434	<.0001		

^a^The phenotypic traits: Growing degree days to heading (GDDTH), growing degree days to maturity (GDDTM), individual stem dry weight at the peak of water-soluble carbohydrates (stem DW), water-soluble carbohydrates accumulation per growing degree days (WSC accumulation per shoot/GDD), and water-soluble carbohydrates remobilization per growing degree days an (WSC remobilization per shoot/GDD).

^b^d.f., Degree of freedom.

^c^MS, Mean of square.

^d^bsh^2^, Broad sense heritability.

Across all environments, stem characteristics were highly affected by genotype, environment, and their interaction. G × E interactions were significant for all the phenotypic traits at *P<* 0.0001 (**Table 2**). The broad sense heritability (*_bs_h* ^2^) for all the phenotypic traits was less than 0.5 except TGW with (*_bs_h* ^2 ^= 0.64), indicating the effect of environmental factors on the phenotypic traits measured. The heritability of the stem traits (IWW, ID, PL was 0.38, 0.13, and 0.34, respectively). IWW had higher heritability compared with other stem traits. The environment highly affected the accumulation and remobilization of WSC/GDD, with *_bs_h* ^2 ^= 0.13 and *_bs_h* ^2 ^= 0.09, respectively (**Table 2**).

### 3.2 Water soluble carbohydrate dynamics are affected by changing environmental conditions

**Figure 3:**
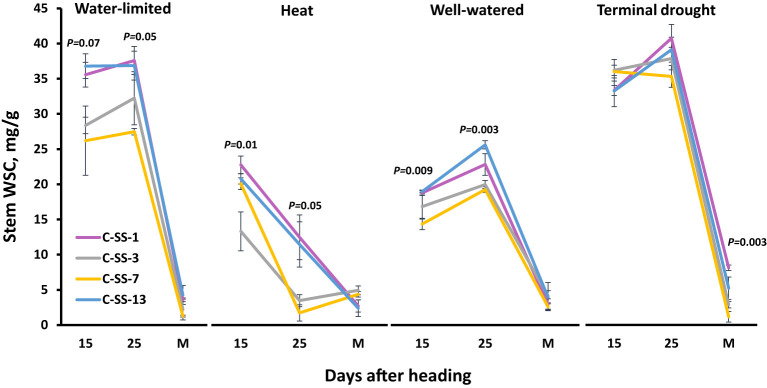
Stem water-soluble carbohydrates (WSC) dynamic during grain filling for two genotypes with high stem WSC (C-SS-1 and C-SS-13) and two with low stem WSC (C-SS-3 and C-SS-7) across four environmental conditions: well-watered (WW), water-limited (WL), heat stress (heat) and terminal-drought (TD). The values are the mean (*n*=4) and standard error of WSC at three time points after heading: 15 and 25 days after heading and at maturity (M).

To better understand the dynamics of WSC, we selected two genotypes with high stem WSC (C-SS-1 and C-SS-13) and two with low stem WSC (C-SS-3 and C-SS-7). These genotypes exhibited stability in WSC accumulation across all four environments (**Figure 3**). Across all environments except heat, the concentration of WSC in the stem reaches the peak 25 days after heading, and translocation to the developing grains occurs from this stage until physiological maturation. Under heat stress, the concentration of stem WSC reached a peak earlier. Under WL, heat, and WW, C-SS-1, and C-SS-13 exhibited significantly higher stem WSC at (*P* = 0.05) than C-SS-3 and C-SS-7 at 15 and 25 DAH. However, there were no significant differences between the 2 groups of genotypes under TD at 15 and 25 DAH, which appeared only at maturity.

**Figure 4:**
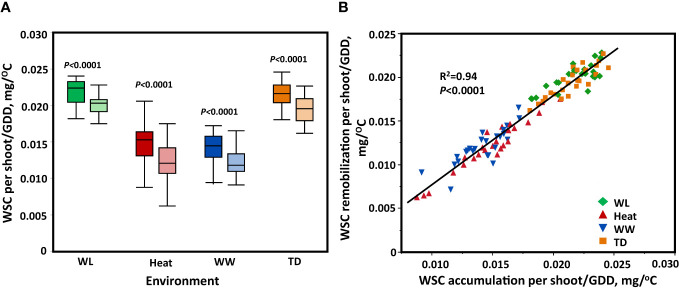
The process of accumulating the WSC and remobilizing it to the grains from heading to maturity between environments. **(A)** Comparison of WSC per shoot/GDD accumulated in the stem up to the peak point (dark color) and WSC/GDD transferred to the spike until maturity (light color) between environments using a t-test. **(B)** Relationship between WSC per shoot/GDD accumulated in the stem up to the peak point, and WSC per shoot/GDD transferred to the spike until maturity. These data represent the rate of accumulation and transfer of WSCper shoot/GDD. Shape and color represent different environments. Well-watered (WW, Blue), water-limited (WL, Green), heat stress (heat, Red), and terminal-drought (TD, Orange).

After analyzing the dynamics of WSC in the stem, WSC was estimated per growing degree days (GGD) in each environment separately. A highly significant variation (*P* < 0.0001) among the genotypes for both WSC accumulation and remobilization per GDD was found in all environments. The accumulation of WSC/GDD under water-limited stress and the terminal-drought was significantly higher (*P *< 0.0001) compared to the heat and well-watered. The remobilization of WSC/GDD was significantly higher (*P *< 0.0001) under water-limited stress and terminal-drought stress compared to heat and well-watered conditions (**Figure 4A**). The result of ANOVA shows that genotype, environment, and their interaction were significant for WSC accumulation and remobilization/GDD (*P* < 0.0001, *P *< 0.0001, respectively). We compared the concentration of WSC accumulated in the stem per GDD up to the peak point (WSC accumulation/GDD), and the amount of sugars transferred to the spike until maturity (WSC remobilization/GDD). A significant positive correlation was found (R^2 ^= 0.94, *P* < 0.0001) across all environmental conditions (**Figure 4B**).

### 3.3 Water-soluble carbohydrates contribute to grain filling under stress conditions

**Figure 5:**
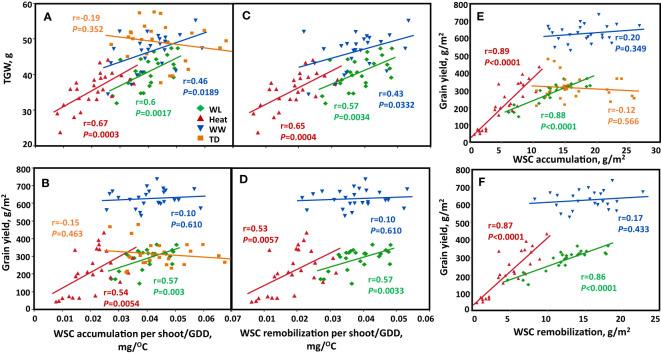
Correlation between stem-WSC accumulation and remobilization ratio (WSC per shoot/GDD) with TGW and GY. **(A, B)** WSC accumulation per shoot/GDD, **(C, D)** WSC remobilization ratio (WSC per shoot/GDD). **(E, F)** WSC accumulation and remobilization per unit area and GY. Correlations are calculated based on genotype mean (*n*=25) across four environmental conditions: Well-watered (WW, Blue), water-limited (WL, Green), heat stress (heat, Red), and terminal-drought (TD, Orange).

In general, high SWSC lines attained high grain size. When we examined the contribution of the quantity of stem WSC to TGW and GY; a significant positive relationship (r = 0.6, *P* = 0.002; r = 0.67, *P* = 0.001; r = 0.46, *P* = 0.019) was found between the quantity of WSC accumulation/GDD and TGW (**Figure 5A**) under water-limited, heat, and well-watered, respectively. We assessed the association between WSC remobilization/GDD and TGW, and a significant positive association was found between the quantity of WSC remobilization/GDD and TGW for heat, water-limited, and well-watered (r = 0.65, *P* = 0.0004); r = 0.57, *P* = 0.0034; and r = 0.43, *P* = 0.0332, respectively; **Figure 5B**). We assessed the effect of the quantity of WSC accumulation and remobilization/GDD on grain yield. A significant positive association between the quantity of WSC accumulation/GDD and GY was found for water-limited and heat (r = 0.57, *P* = 0.003 and r = 0.54, *P* = 0.0054, respectively; **Figure 5C**). Additionally, the quantity of WSC remobilization/GDD also had a significant positive effect on the grain yield under WL and heat environments (r = 0.57, *P* = 0.0033 and r = 0.53, *P* = 0.0057, respectively; **Figure 5D**).

We checked the associations between WSC accumulation per unit area with GN and GY, We found a significant positive relationship (r = 0.88, *P *< 0.0001; r = 0.80, *P *< 0.0001) between WSC accumulated per unit area (g/m^2^) and GN per m^2^ under heat and WL respectively (**Supplementary Figure S4C**). WSC accumulated per unit area was positively associated with GY under heat (r = 0.89, *P *< 0.0001), and WL (r = 0.88, *P *< 0.0001) (**Figure 5E**). WSC remobilization per unit area correlated positively with GN (r = 0.85, *P *< 0.0001; r = 0.79, *P *< 0.0001) under heat and WL respectively (**Supplementary Figure S4D**). This contributed to GY (r = 0.87, *P *< 0.0001) under heat, and (r = 0.86, *P *< 0.0001) under WL (**Figure 5F**). The correlation was not significant in the WW environment for both WSC accumulated and remobilized per unit area with GY and GN. In the terminal-drought environment, we had missing data on stem dry weight at maturity; therefore, we could not check the association between TGW, GY, GN, and the quantity of WSC remobilized and WSC remobilized per unit area from the stem to the spike under the terminal-drought environment.

### 3.4 Internode diameter and peduncle length contribute to water-soluble carbohydrates under stress conditions with no trade-off in grain yield

**Figure 6:**
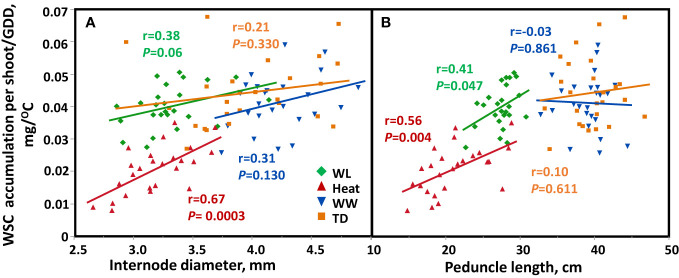
Correlation between **(A)** internode diameter or **(B)** peduncle length and stem water soluble (WSC) accumulation ratio (WSC per shoot/GDD) based on genotype mean (*n*=25) across four environmental conditions: Well-watered (WW, Blue), water-limited (WL, Green), heat stress (heat, red), and terminal-drought (TD, Orange).

Assessment of the stem traits’ contribution to crop growth under four environments showed a significant positive correlation between stem internode diameter with the quantity of WSC accumulation/GDD in the stem (r = 0.67, *P* = 0.0003 and r = 0.38, *P* = 0.060 under heat and WL, respectively; **Figure 6A**), under WW and TD the correlation was not significant. In addition, stem diameter was also positively correlated with WSC accumulation per unit area under heat stress (r = 0.64, *P* = 0.0005, **Supplementary Figure S4A**). A positive correlation was found between peduncle length and the quantity of WSC accumulation/GDD (r = 0.41, *P* = 0.047, and r = 0.56, *P* = 0.0038, for WL and heat, respectively; **Figure 6B**). Peduncle length was positively associated with WSC accumulated per unit area (r = 0.74, *P* < 0.0001) under heat, and WL (r = 0.49, *P* = 0.013, **Supplementary Figure S4B**).

For stem solidness, a negative correlation was found between the quantity of WSC accumulation/GDD and stem solidness (r = -0.4, *P* = 0.043 and r = -0.39, *P* = 0.05 for WL and heat, respectively) (**Supplementary Figure S2A**).

The correlation was not significant under WW and TD environments. However, at 15DAH, the internode diameter correlated positively with WSC under TD environments, heat, and water-limited (**Supplementary Figure S2B**).

**Figure 7:**
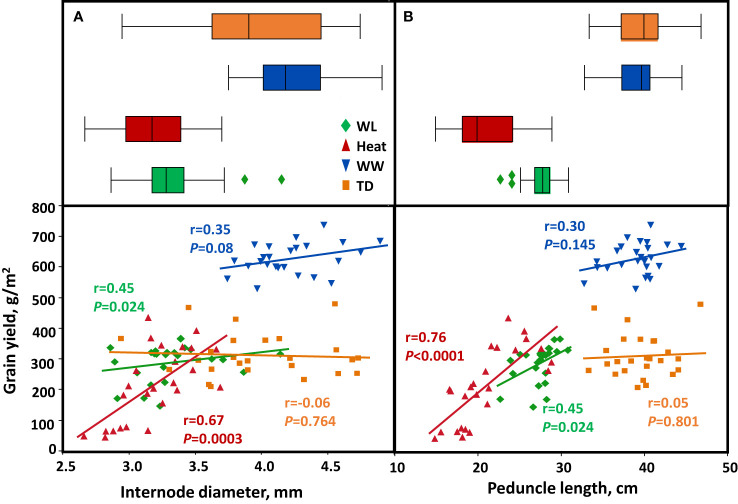
Correlation between stem characters and grain yield. **(A)** internode diameter, **(B)** peduncle length. Correlations are calculated based on genotype mean (*n*=25) across four environmental conditions: Well-watered (WW, Blue), water-limited (WL, Green), heat stress (heat, Red), and terminal-drought (TD, Orange).

The stem characteristics (internode diameter and peduncle length) contributed to the grain filling under water-limited and heat stress. A highly significant positive relationship between peduncle length and TGW under heat (r = 0.7, *P *= 0.0001), WL (r = 0.38, *P* = 0.06), and TD (r = 0.5, *P* = 0.01) environments (**Supplementary Figure S3B**). In addition, peduncle length was positively associated with GY under heat and WL environments (r = 0.76, *P *< 0.0001 and r = 0.45, *P* = 0.002, respectively; - **Figure 7B**). On the other hand, internode diameter showed a strong correlation with TGW under heat stress (r = 0.8, *P *< 0.0001) and WW (r = 0.48, *P* = 0.015) (**Supplementary Figure S3A**), which contributed to increased GY (r = 0.67, *P* = 0.0003 and r = 0.35, *P* = 0.08, respectively) (**Figure 7A**). WL and heat environments negatively affected crop productivity and structural stem biomass, as expressed in lower ID and short PL (**Figures 7A, B**). The PL was significantly different between environments (*P *< 0.0001), with the lowest value recorded under heat (20.0 cm) compared to TD (39.6 cm). Peduncle, SWSC content, and remobilization were positively associated with significantly enhanced grain filling (TGW and GY) under WL and heat conditions.

In WW and TD environments, there was no trade-off between increased crop investment in internode diameter and peduncle length and GY **Figures 7A, B**).

We checked the association between ID, PL, and grain number per m^2^. Across environments, there was no trade-off between ID, PL, and grains per m^2^. Under heat stress internode diameter correlated positively (r = 0.60, *P* = 0.013) with grains per m^2^. On the other hand, peduncle length was positively associated with grain number per m^2^ (r = 0.71, *P *< 0.0001 and r = 0.34, *P* = 0.09) under heat and WL environments respectively (**Supplementary Figures S3C, D**).

## 4 Discussion

To face the challenges of increasing wheat production under the current and projected climate change, wheat research, and breeding requires a comprehensive exploration of potential genetic resources and an in-depth understanding of their underlying environmental adaptations. Blum (1998) suggested that improving stem reserve storage and remobilization can contribute to grain filling under terminal-drought conditions. Moreover, crop investment in stem solidness is associated with high stem WSC and can facilitate improved grain filling under water-limited conditions (Saint Pierre et al., 2010). Here, we want to re-evaluate these findings in light of the contribution of other stem characteristics (including water-soluble carbohydrate, solidness, diameter, and peduncle length) to yield components under various environmental conditions (water-limited, heat stress, and terminal-drought).

### 4.1 Changing environmental conditions cause stem traits’ phenotypic instability

Adaptation to drought and heat stress involves a suite of traits, including stem traits. Understanding how stem traits interact with different environmental conditions is crucial in developing an ideal wheat stem ideotype that can adapt to changing climate conditions. In the current study stem characteristics were highly affected by the genotype, environment, and their interaction (**Figure 2**).

The broad sense heritability (*_bs_h* ^2^) for stem traits (IWW, ID, and PL) was less than 0.5 (**Table 1**), indicating an effect of environmental cues. Stem solidness exhibited the highest values under WL (65%) and the lowest under TD (43%) (**Table 2**). Similarly, Hayat et al. (1995) showed that the expression level of stem solidness is affected by the environment, and stems tend to be more solid when plants get exposed to drought or high temperatures during stem elongation. However, unlike Saint Pierre et al. (2010), our multi-environment trials showed that an increase in stem solidness was negatively correlated with the amount of stem WSC (**Supplementary Figure S2A**). The stem solidness may combine with yield-attributing traits differently in different genetic backgrounds. Bainsla et al. (2020) found that in a segregating population (HD2967 × RAJ 4422), the frequency of favorable segregants with optimum plant height, increased spikelet number, and more stem solidness were relatively high compared to other parental combinations. WL and heat stress conditions had a detrimental impact on structural stem biomass, which was evident in reduced ID and PL under WL and heat (**Figures 7A, B**).

Our results are in agreement with the finding reported by Sallam et al. (2015), who observed a substantial decrease in stem diameter as a result of heat stress, which was positively and significantly associated with a reduction in TGW and grain yield per spike. In addition (Ehdaie et al., 2006a) reported that internode length and weight were reduced under drought. These results are similar to the findings of Ahmed et al. (2014), who stated that drought stress reduced stem diameter (25%) and heat stress by 6%. The combined drought and heat stress amounted to a 31% reduction in stem diameter. WL and heat stress reduced stem diameter by 21.5 and 24.5% and PL by 29 and 46%, respectively. This reduction in crop stem structural biomass could probably be explained by the lower availability of resources under stress conditions (WL and heat). Still, these results highlight the necessity and significance of improving stem diameter and peduncle length, particularly under stressful conditions.

### 4.2 The peak of water-soluble carbohydrate accumulation in the stem is environmentally dependent

The maximum WSC mg g^-1^ accumulated occurred approximately 25 days after heading across all environments except heat (**Figure 3**), with the highest value recorded in WL and TD environments (**Figure 4A**). These results are in accordance with Liu et al. (2020) and Ruuska et al. (2006), who found that stem WSC accumulation increased under drought conditions, and the remobilization to the spike occurs from this stage until physiological maturation. Our results also indicate that WSC accumulation increases under WL and TD compared to WW and heat stress.

Depending on the level of stress, the peak can be reached much earlier), as it was found from the WSC dynamic under heat (**Figure 3**). Three out of four genotypes, the peak can also be reached earlier (before 15 days after heading) with the WL environment. WL would reduce the period of WSC accumulation after anthesis, allowing for earlier remobilization. Wardlaw and Willenbrink (1994) found in a pot experiment that under non-stress conditions, plants showed a maximum accumulation of WSC between 20 to 25 days after anthesis, and the mobilization varied between cultivars. In our field-based study, WSC results revealed a similar trend, including significant (*P <* 0.0001) differences between genotypes in both accumulation and remobilization, which were highly correlated (**Figure 4B**). In contrast, Yáñez et al. (2017) found that maximum accumulation of WSC occurred 10–20 days after anthesis under water stress and full irrigation (50% and 100% field capacity, respectively).

WSC accumulation (peak) correlated positively with WSC remobilization (R^2 ^= 0.94, *P* < 0.0001) across all environments (**Figure 4B**). Zamani and Nabipour (2014) found similar results under non-stress conditions (R^2 ^= 0.69, *P <* 0.001) and heat stress conditions (R^2 ^= 0.72, *P *< 0.001). These findings support Blum’s et al. (1994) findings that a cultivar with more stem reserves remobilized two to three times more stem reserves than a cultivar with less stem reserves. These results indicate that regardless of environmental conditions, the accumulation of WSC is correlated with WSC remobilization.

Therefore, the WSC accumulation ratio could be considered a suitable trait for breeders when selecting genotypes with maximum storage capacity and remobilization of stem reserves. The remobilization rate suggests that stem WSC had been mobilized toward the developing grain, highlighting WSC as a potential trait for selection in pre-breeding programs that focus on grain-filling resilience in wheat under stress conditions.

### 4.3 Water limitation accelerates the remobilization of water-soluble carbohydrates enhancing grain filling

During grain-filling, WSC remobilization into the grain is one of the physiological adjustments that wheat plants use to adapt to stressful environmental conditions. In this study, we checked the contribution of stem WSC to grain filling under WW, WL, heat, and TD. We found that WSC stem reserve utilization by the developing grains depends on the environment and remobilization ability during grain filling. The remobilization rate of WSC was high under WL and TD compared to the heat and WW environments (**Figure 4A**).

This finding is consistent with the other reports, which found that water deficit during grain filling of wheat facilitates the mobilization efficiency of WSC in the stem through the regulation of enzymes involved in fructan and sucrose metabolism (Yang et al., 2004; Hou et al., 2018; Islam et al., 2021; Morgun et al., 2022). Liu et al. (2020) also stated that drought stress curtailed the peak of WSC levels and depressed WSC accumulation but significantly accelerated the remobilization of pre-anthesis WSC during the grain filling. These results indicate that the highest remobilization of WSC under WL and TD is due to the decline in assimilating sources from photosynthesis, which leads the plants to depend on stem storage.

Previous studies have found that WSC contributes to grain filling (grain weight per spike) under water deficit (r = 0.5, *P *< 0.001); heat (r = 0.72, *P *< 0.001), and combined water deficit and heat stress (r = 0.67, *P *< 0.001) (Gurumurthy et al., 2023). In the present study, we found similar results where both WSC accumulation ratio positively correlated with TGW and GY under heat and WL conditions (**Figures 5A, B**), and the remobilization ratio with TGW and GY under heat and WL (**Figures 5C, D**). A similar observation was reported by (Fu et al., 2020), who found a positive correlation between WSC and TGW in the WL environment. Other studies also found that WSC correlated positively with grain weight and grain yield under rain-fed conditions (McIntyre et al., 2012); and grain yield in droughted field conditions (Ehdaie et al., 2008).

In the present study, WSC accumulated and remobilized per unit area were positively associated with grain number per m^2^ under heat and WL (**Supplementary Figures S4C, D**) which contributed to GY under heat and WL environments (**Figures 5E, F**). These findings indicate the importance of this trait to the grain-filling process under water-limited, drought, and heat stress conditions. Furthermore, these relationships illustrate that genotypes with higher stem WSC are likely to be less affected by WL and heat stress compared to genotypes that accumulate lower stem WSC.

Our results show that the lines that accumulate more WSC (e.g., C-SS-1; C-SS-13, C-SS-17) also remobilize more WSC (*P <* 0.0001), benefitting in higher grain size and yield compared to low WSC lines (e.g., C-SS3; C-SS-7). In addition, these lines exhibit a low reduction in grain number per m^2^, and GY under WL, heat, WW, and TD environments, respectively (**Supplementary Table S2**). These results are consistent with the findings reported by Hou et al. (2018) and Liu et al. (2020), who found that high WSC lines had a greater amount of WSC and higher remobilization efficiency in addition to high grain yield compared to the lines with low WSC under drought stress. In addition, similar to our results, Rebetzke et al. (2008) reported that lines with high WSC had more grain number, yield, and kernel weight or size compared with genotypes with low WSC. This is also consistent with the finding of Islam et al. (2021), who observed that BARI Gom 24 (a line with high stem reserves) showed high remobilization and higher grain yield under drought stress compared to Kanchan and BARI Gom 25 (lines with low stem reserves).

In our study, the correlation between WSC remobilization and TGW, GN, GY under WL, and heat stress shows that the genotypes that exhibit higher WSC remobilization also maintain a high sink strength under stress conditions. Therefore, selecting genotypes with a higher stem WSC accumulation and remobilization ratio could be utilized as a strategy to buffer grain growth and development under WL and heat stress conditions.

### 4.4 Increased internode diameter and peduncle length are associated with water-soluble carbohydrates and grain size with no yield penalty across environments

Although stem WSC plays a role in grain filling, it is a complex trait to measure and requires a substantial investment of time and money. Consequently, there is a need to identify easily measurable traits correlated with WSC which can be used as an effective selection tool for this trait. In our study, we aimed to determine which stem traits positively correlate with WSC, thereby serving as suitable proxies for selecting high-stem WSC. Our results show that a positive correlation between structural stem diameter (measured at the 2^nd^ internode) did result in a higher amount of WSC in the stem under heat and WL conditions (**Figure 6A**), similar to the results found by (Ehdaie et al., 2006b). In addition, stem diameter was correlated with WSC accumulation per unit area under heat stress (**Supplementary Figure S4A**). Stem diameter was also positively correlated with TGW (**Supplementary Figure S3**), and GY (**Figure 7A**) under heat and WL environments. These results are in agreement with the finding of Ahmed et al. (2014), who found a significant positive correlation between stem diameter with TGW (r = 0.56, 0.53 and 0.56 *P *< 0.01) and grain yield per spike (r = 0.4, r = 0.4 and 0.5, *P *< 0.05) under favorable, drought and drought + heat stress environments respectively. In the current study, we found that internode diameter (measured at the second internode) was correlated positively with grain number per m^2^, under heat stress, while under WL, TD, and WW environments there is no trade-off, between internode diameter and GN (**Supplementary Figure S3C**), unlike the results reported by Sierra-Gonzalez et al. (2021) who found that internode 3 (below the penultimate internode) and internode 2 (below the peduncle, Rivera-Amado et al., 2019) dry matter partitioning were negatively associated with GN.

Sallam et al. (2015) also observed a highly significant correlation (r = 0.56, under drought and 0.66 under drought + heat) between stem diameter and TGW. The correlation between stem diameter and WSC, TGW, and GY under WL and heat confirms the strong relationship between this trait and yield attributes under stressful environmental conditions. Therefore, stem diameter is important in sustaining grain filling under stressful conditions due to the ability to store stem reserves. In addition, we found that increased investment in peduncle length (*_bs_h* ^2^ = 0.34) results in higher WSC accumulation under stress (**Figure 6B**). Moreover, it was also positively associated with WSC accumulated per unit area (**Supplementary Figure S4B**), TGW under heat, WL, and TD environments (**Supplementary Figure S3B**); and GY under heat and WL conditions (**Figure 7B**), while under WW, the correlation was not significant. These results are in accordance with previous findings, which showed that the highest levels of WSC are located between the peduncle and penultimate internode (Zhang et al., 2015); and upper internodes during the early period of grain filling (Morgun et al., 2022).

Peduncle length and penultimate internode length were also positively correlated with a high number of spikelets, which is an important contributor to grain yield in different genetic combinations (Bainsla et al., 2020). In our study peduncle length was positively associated with grain number per m^2^ under heat and WL conditions (**Supplementary Figure S4D**). A positive correlation (r = 0.49) between the total amount of WSC in the upper internode and grain yield under water deficiency was found in the study done by Saint Pierre et al. (2010) evaluating 36 wheat genotypes differing in stem solidness, this indicates a positive contribution of WSC to the grain yield. The contribution of peduncle length to WSC and its correlation between TGW, GN, and GY shows how important this trait is in sustaining grain filling under stressful conditions mainly due to its ability to store more stem reserves. Therefore, ID and PL play an important role in grain filling under stressful conditions, and selecting genotypes with wider diameters and long peduncles can result in high WSC in addition to GY under water-limited and heat stress. However, more research is needed to validate these trends.

Internode diameter and peduncle length were correlated positively with stem dry weight at the peak of WSC under heat, WL, and WW environments, furthermore, stem dry weight was positively correlated with WSC across all environments (results not shown). Both stem dry and water-soluble carbohydrates contributed significantly to the accumulation of WSC across environments. Internode diameter was positively correlated with the concentration of WSC (%) in the stem under heat, but the correlation was not significant under WL, TD, and WW. On other hand, PL correlated negatively with WSC concentration under WW environments (results not shown).

In the current study, under WL and TD, there was no trade-off between grain yield and increased investment in ID and PL (**Figures 7A, B**). However, under WL and heat stress conditions, there was a significant increase in grain (**Figures 7A, B**). Therefore, stem diameter and peduncle length could be combined in a single plant ideotype to maximize WSC reserves targeting TGW and yield improvement under water-limited and heat-stress conditions. For example, C-SS-1 and C-SS-13 with significant wide diameter (*P* < 0.0001) and long peduncle (*P *< 0.0001) (**Supplementary Table S2**) had higher WSC accumulation, WSC remobilization, TGW and GY compared to C-SS-3 and C-SS-6 across all environments. The reduction in stem characters (ID and PL) and its relationship with the decrease in GY, GN, and TGW due to drought and heat stress demonstrate the importance of the stem characters in sustaining grain size and GY under stress due to their ability to store WSC supporting post-anthesis photosynthesis.

## 5 Conclusions and application for breeding

The results of our study show that water-limited and heat stress conditions negatively affected both biomass and grain yield, but also structural stem biomass as expressed in lower internode diameter and peduncle length. Nevertheless, internode diameter, peduncle length, stem WSC accumulation, and remobilization were positively associated and significantly enhanced grain filling under water-limited and heat stress conditions. It is worth noting that there was no trade-off between grain yield and increased crop investment in both internode diameter and peduncle length under optimal conditions and terminal-drought. However, under water-limited and heat stress conditions increased crop investment in stem diameter and peduncle length promotes higher grain yield.

WSC content has not been widely used as a direct breeding objective (Ruuska et al., 2006), possibly due to the combined limitations of WSC phenotyping and environmental variability. Our results show that genotypes with high SWSC content also have high WSC remobilization. However, the maximum accumulation (the peak) depends on environmental conditions, as also observed in other reports (Foulkes et al., 2002; Shearman et al., 2005; Ruuska et al., 2006) where under severe heat stress the peak of WSC accumulation can be reached early. In the pre-breeding context, we showed that stem characters (stem diameter and peduncle length) play a key role in sustaining grain filling under stressful conditions and can be good indicators (proxies) of high WSC under water-limited and heat stress. We suggest that crop investment in structural biomass traits might be a worthwhile strategy to offset TGW reduction under water-limited and high-temperature conditions in semi-dwarf spring wheat.

## Data availability statement

The raw data supporting the conclusions of this article will be made available by the authors, without undue reservation.

## Author contributions

SN: Conceptualization, Data curation, Formal analysis, Methodology, Writing – original draft, Writing – review & editing, Validation, Visualization, Investigation. MZ: Investigation, Data curation, Writing – original draft, Writing – review & editing. KN: Writing – original draft, Writing – review & editing, Data curation, Investigation. DB: Data curation, Writing – original draft, Writing – review & editing, Conceptualization, Methodology, Visualization, Resources. NB: Writing – original draft, Writing – review & editing, Data curation, Methodology, Conceptualization, Investigation. FP-C: Data curation, Methodology, Writing – original draft, Writing – review & editing, Conceptualization, Validation, Investigation. MR: Conceptualization, Data curation, Methodology, Writing – original draft, Writing – review & editing, Visualization, Investigation. ZP: Conceptualization, Investigation, Visualization, Writing – original draft, Writing – review & editing, Supervision. RB-D: Conceptualization, Investigation, Project administration, Resources, Supervision, Validation, Visualization, Writing – original draft, Writing – review & editing, Methodology, Data curation.
