# Supplementary material for: Stem traits promote wheat climate-resilience
Source: Front Plant Sci. 2024 Jul 25;15:1388881. doi: 10.3389/fpls.2024.1388881 (PMC11308436; doi:10.3389/fpls.2024.1388881)
Supplement: Supplementary file 1 [file DataSheet_1.docx]

Supplementary Material


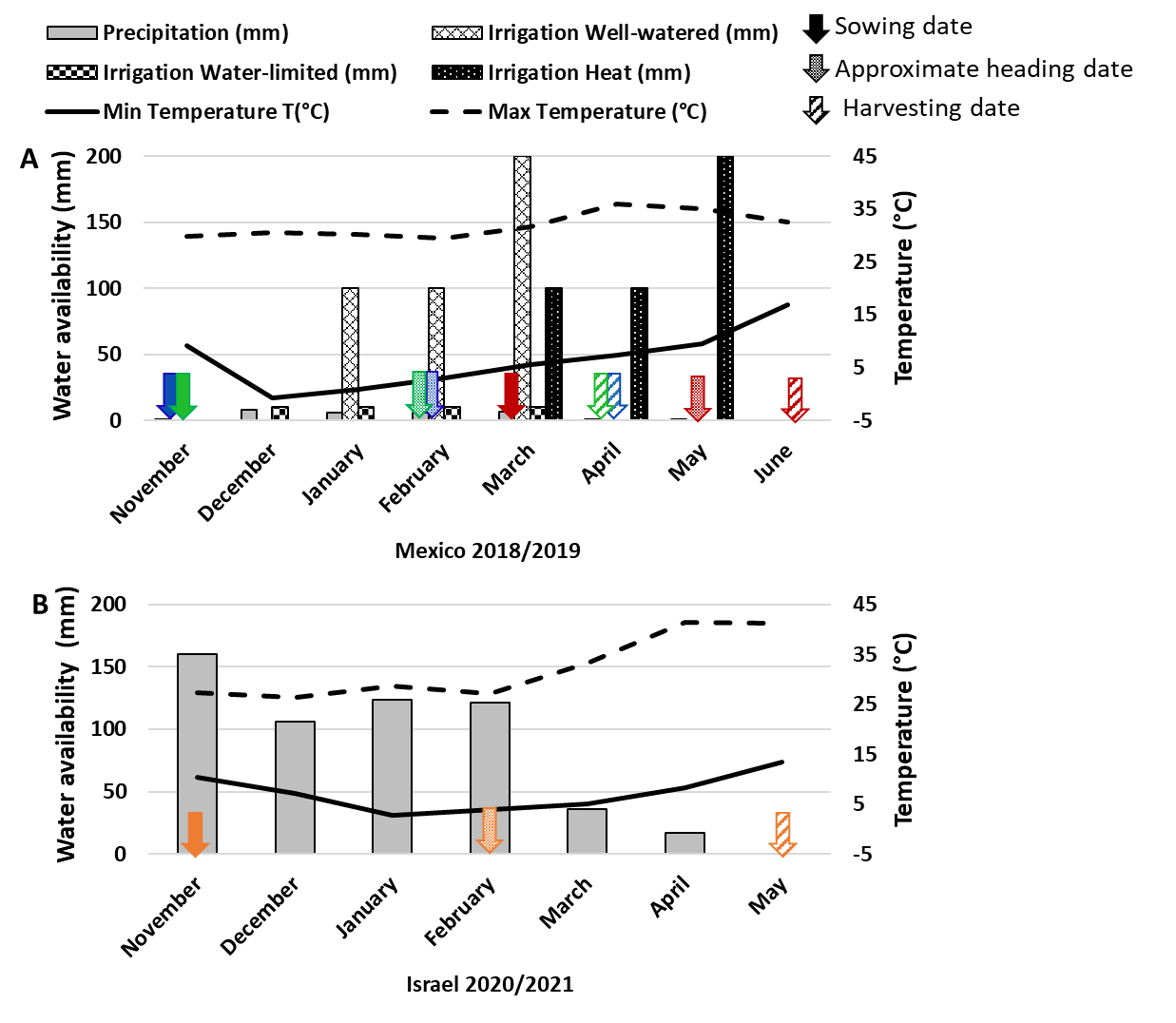


**Supplementary Figure S1.** Seasonal temperature, precipitation, and supplemental irrigation of the four environments studied in (**A**) Mexico, and (**B**) Israel. The color represents the environment. Well-watered (Blue), water-limited (Green), heat (Red), and terminal-drought (Orange). The full arrow indicates the sowing time, and the hollow arrow with dots indicate heading date, and the hollow arrow with lines indicates the harvesting time.

**
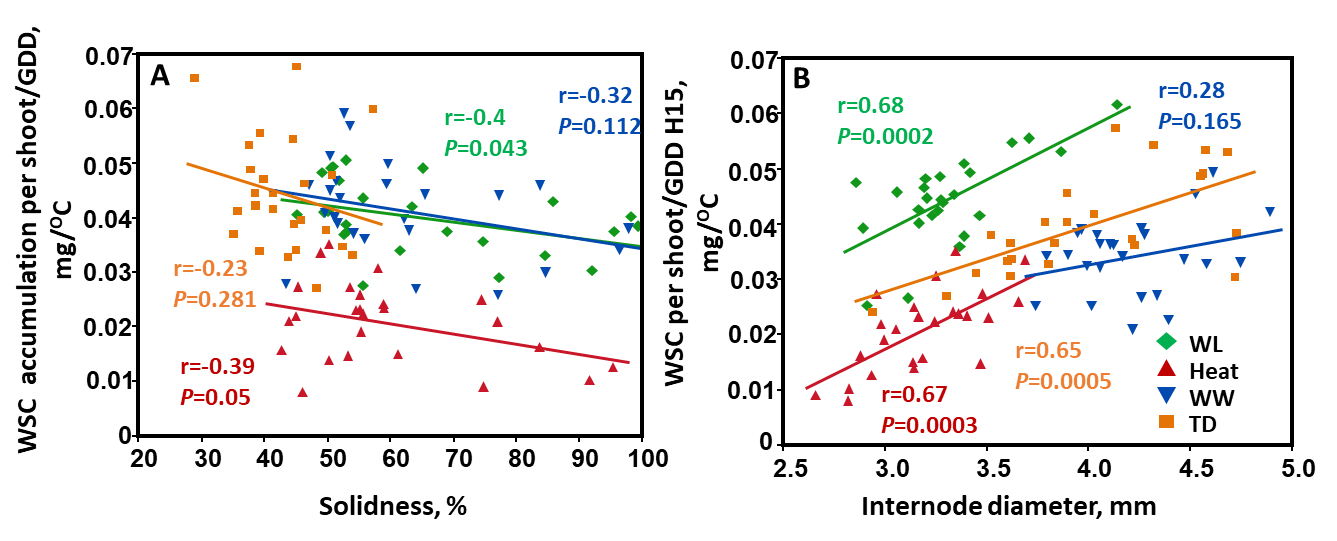
Supplementary Figure S2.** Correlation between (**A**) stem solidness and water-soluble carbohydrate accumulation per growing degree days (WSC accumulation per shoot/GDD), **(B)** internode diameter, and water-soluble --carbohydrate per growing degree days at 15 days after heading (WSC per shoot/GDD H15) based on genotype mean (*n* = 25) across four environmental conditions: well-watered (WW, Blue), water-limited (WL, Green), heat stress (heat, Red), and terminal-drought (TD, Orange).

**
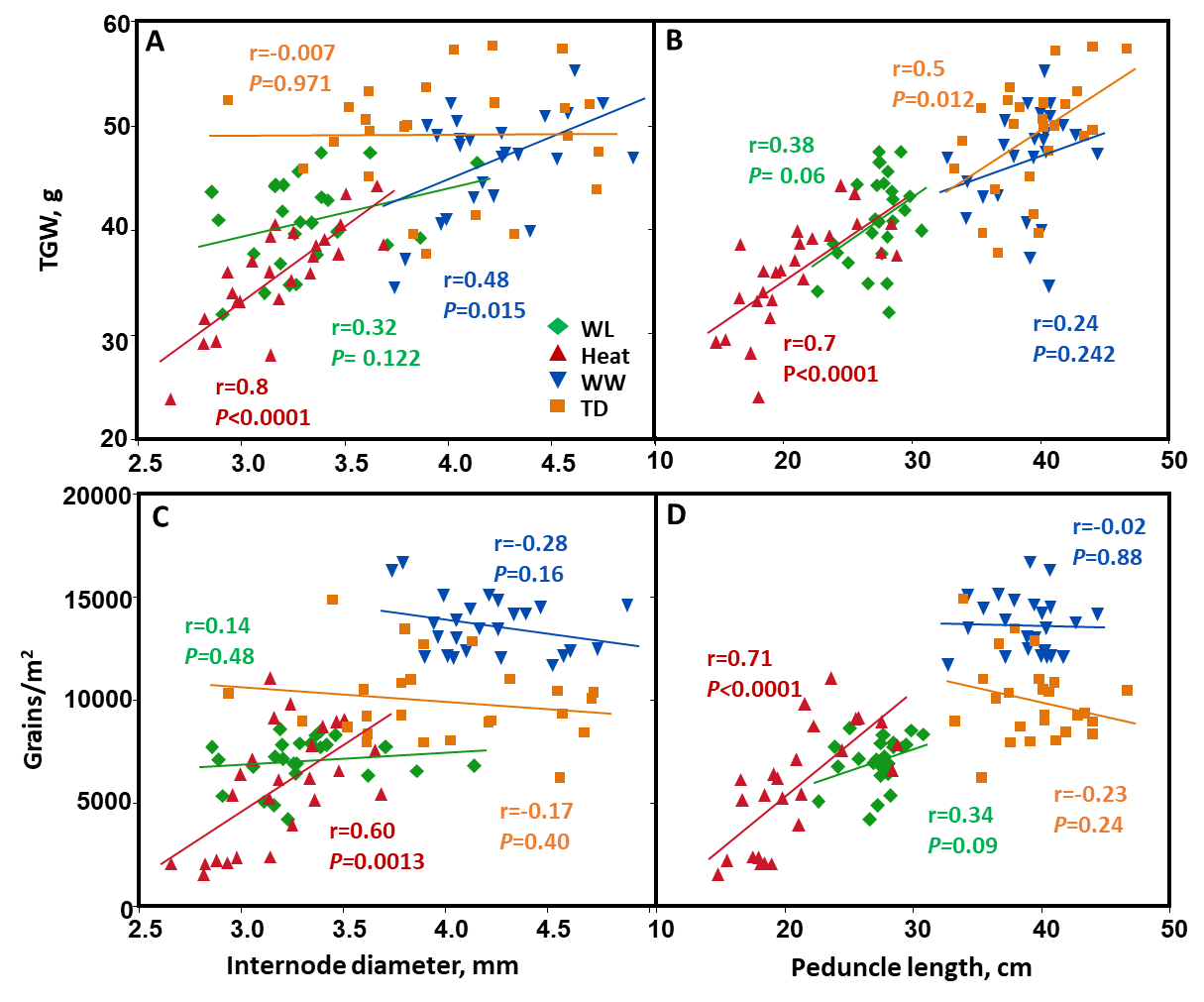
Supplementary Figure S3.** Correlation between (**A-B**) internode diameter or (**C-D**) peduncle length and (thousand-grain weight (TGW) or grain per m^2^. Data is mean (*n*=25) across four environmental conditions: well-watered (WW, Blue), water-limited (WL, Green), heat stress (heat, Red), and terminal-drought (TD, Orange).

**
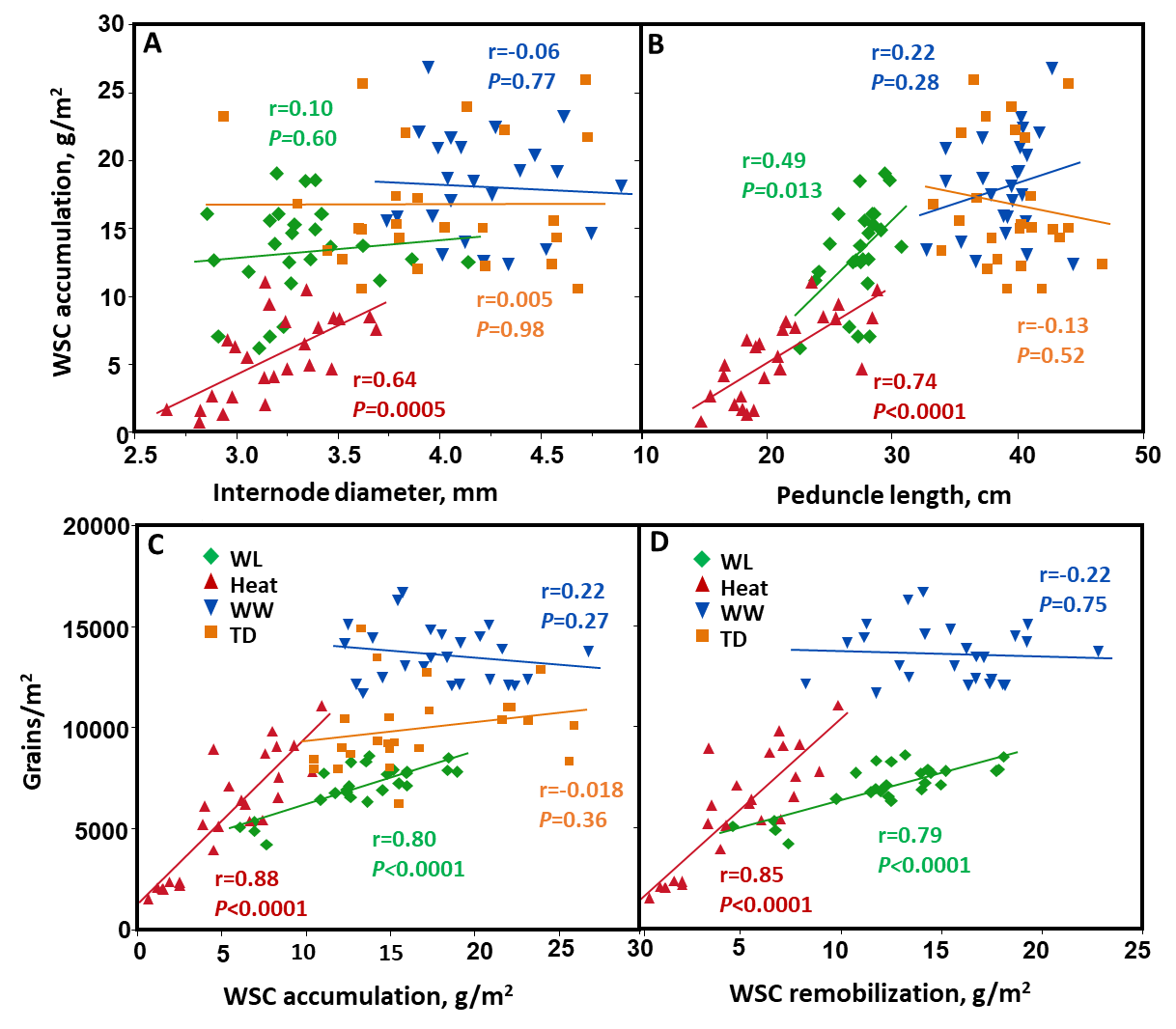
Supplementary Figure S4.** Correlation between (**A**) internode diameter or (**B**) peduncle length and water-soluble carbohydrates (WSC) accumulation per m^2^. (**C**) WSC accumulation per m^2,^ (**D**) WSC remobilization per m^2^ and grain per m^2^. Data is mean (*n*=25) across four environmental conditions: well-watered (WW, Blue), water-limited (WL, Green), heat stress (heat, Red), and terminal-drought (TD, Orange).
