# Supplementary material for: Stem traits promote wheat climate-resilience
Source: Front Plant Sci. 2024 Jul 25;15:1388881. doi: 10.3389/fpls.2024.1388881 (PMC11308436; doi:10.3389/fpls.2024.1388881)
Supplement: Supplementary file 3 [file Presentation_1.pptx]

## Slide 1
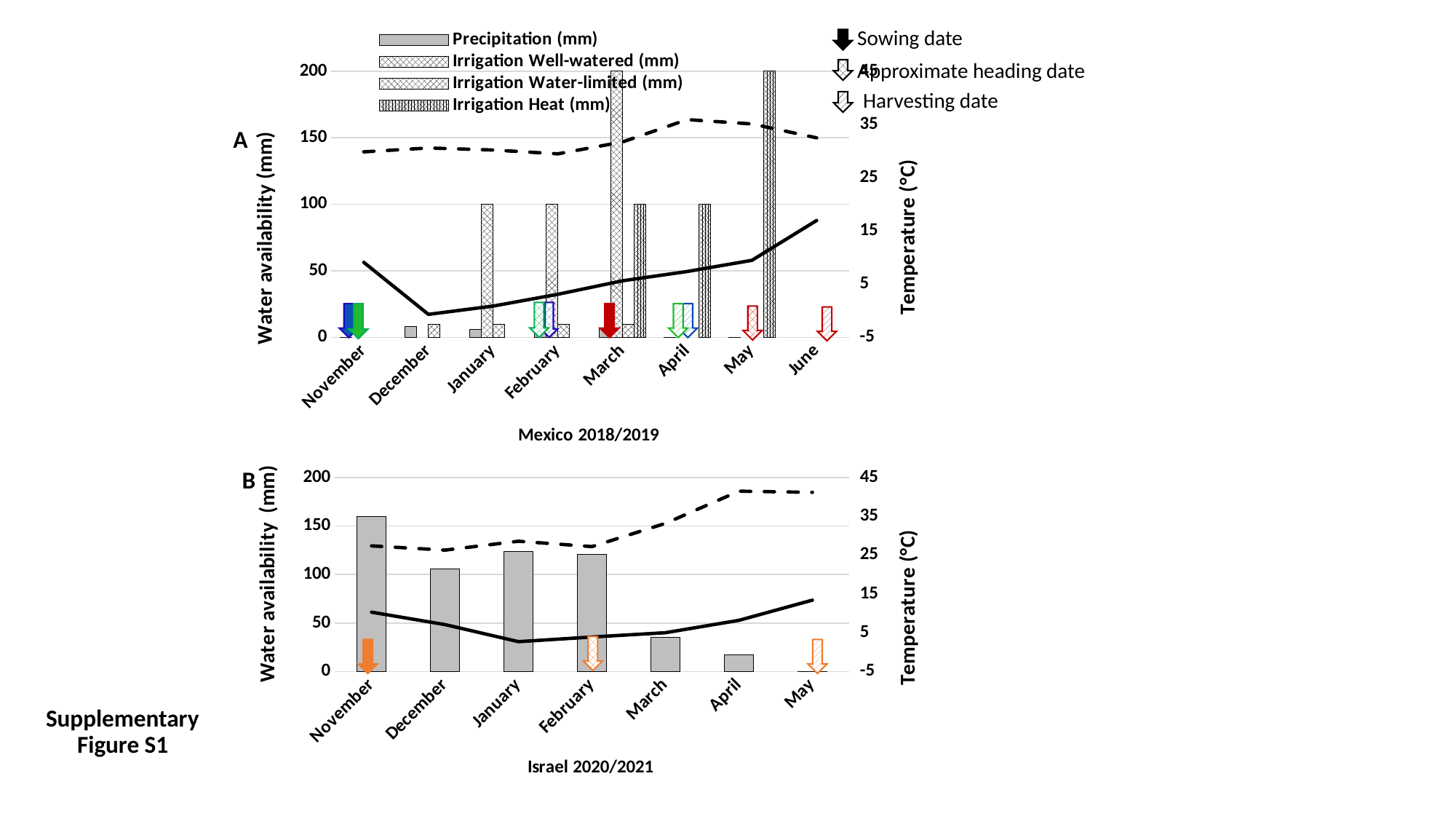

### Chart
| Category | Precipitation (mm) | Irrigation Well-watered (mm) | Irrigation Water-limited (mm) | Irrigation Heat (mm) | Min Temperature T(°C) | Max Temperature (°C) |
|---|---|---|---|---|---|---|
| November | 0.1 | None | None | None | 9.09 | 29.85 |
| December | 7.999999999999999 | None | 10.0 | None | -0.69 | 30.58 |
| January | 5.8999999999999995 | 100.0 | 10.0 | None | 0.87 | 30.2 |
| February | 5.699999999999999 | 100.0 | 10.0 | None | 3.08 | 29.48 |
| March | 6.5 | 200.0 | 10.0 | 100.0 | 5.62 | 31.71 |
| April | 0.1 | None | None | 100.0 | 7.36 | 35.91 |
| May | 0.1 | None | None | 200.0 | 9.46 | 35.09 |
| June | None | None | None | None | 16.96 | 32.48 |
### Chart
| Category | Precipitation (mm) | Min Temperature T(°C) | Max Temperature (°C) |
|---|---|---|---|
| November | 160.0 | 10.3 | 27.4 |
| December | 106.0 | 7.1 | 26.3 |
| January | 124.0 | 2.7 | 28.6 |
| February | 121.0 | 3.9 | 27.2 |
| March | 35.6 | 5.0 | 33.2 |
| April | 17.0 | 8.2 | 41.5 |
| May | 0.0 | 13.4 | 41.2 |A
B
Sowing date
Approximate heading date
Harvesting date
Supplementary Figure S1

## Slide 2
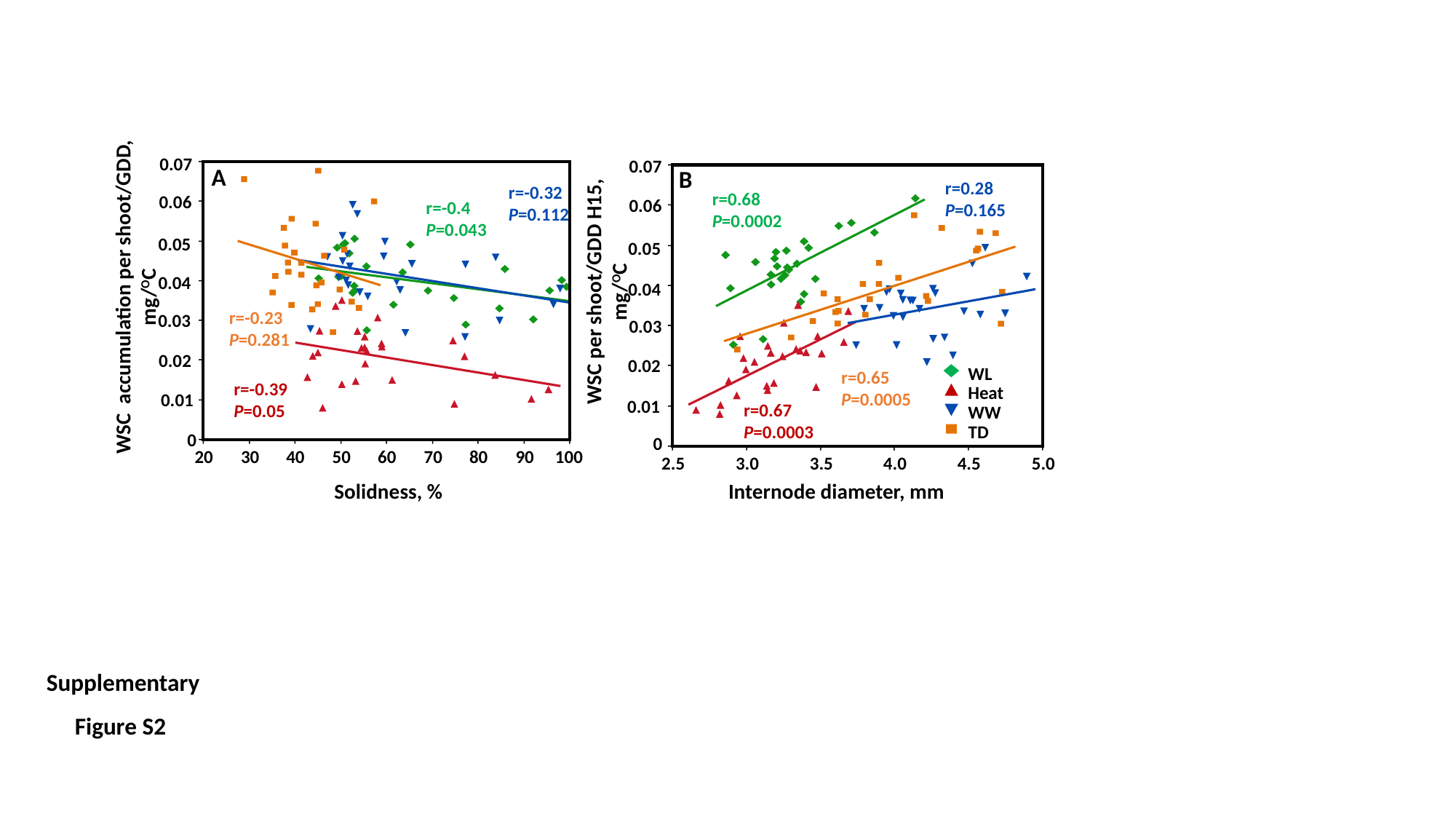

0.07
0.06
0.05
0.04
0.03
0.02
0.01
0
20
30
40
50
60
70
80
90
100
0.07
0.06
0.05
0.04
0.03
0.02
0.01
0
2.5
3.0
3.5
4.0
4.5
5.0
A
B
r=0.28
P=0.165
r=-0.32
P=0.112
r=0.68
P=0.0002
r=-0.4
P=0.043
WSC per shoot/GDD H15, mg/OC
WSC accumulation per shoot/GDD, mg/OC
r=-0.23
P=0.281
WL
Heat
WW
TD
r=0.65
P=0.0005
r=-0.39
P=0.05
r=0.67
P=0.0003
Solidness, %
Internode diameter, mm
Supplementary Figure S2

## Slide 3
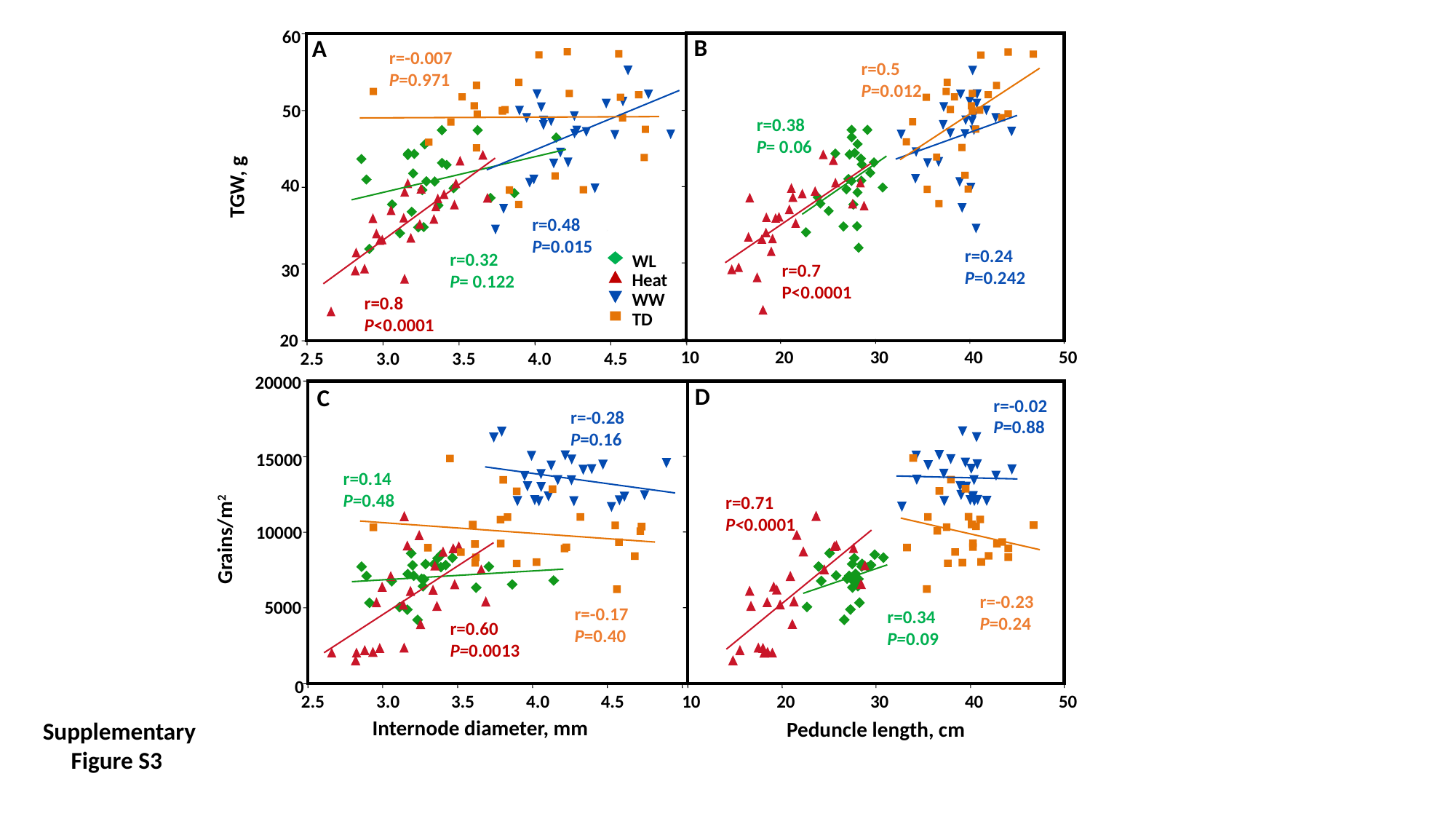

60
50
40
30
20
2.5
3.0
3.5
4.0
4.5
B
TGW, g
A
10
20
30
40
50
r=-0.007
P=0.971
r=0.5
P=0.012
r=0.38
P= 0.06
r=0.48
P=0.015
WL
Heat
WW
TD
r=0.24
P=0.242
r=0.32
P= 0.122
r=0.7
P<0.0001
r=0.8
P<0.0001
Grains/m2
20000
15000
10000
5000
0
2.5
3.0
3.5
4.0
4.5
r=-0.28
P=0.16
r=0.14
P=0.48
r=-0.17
P=0.40
r=0.60
P=0.0013
Internode diameter, mm
10
20
30
40
50
r=-0.02
P=0.88
r=0.71
P<0.0001
r=-0.23
P=0.24
r=0.34
P=0.09
Peduncle length, cm
D
C
Supplementary Figure S3

## Slide 4
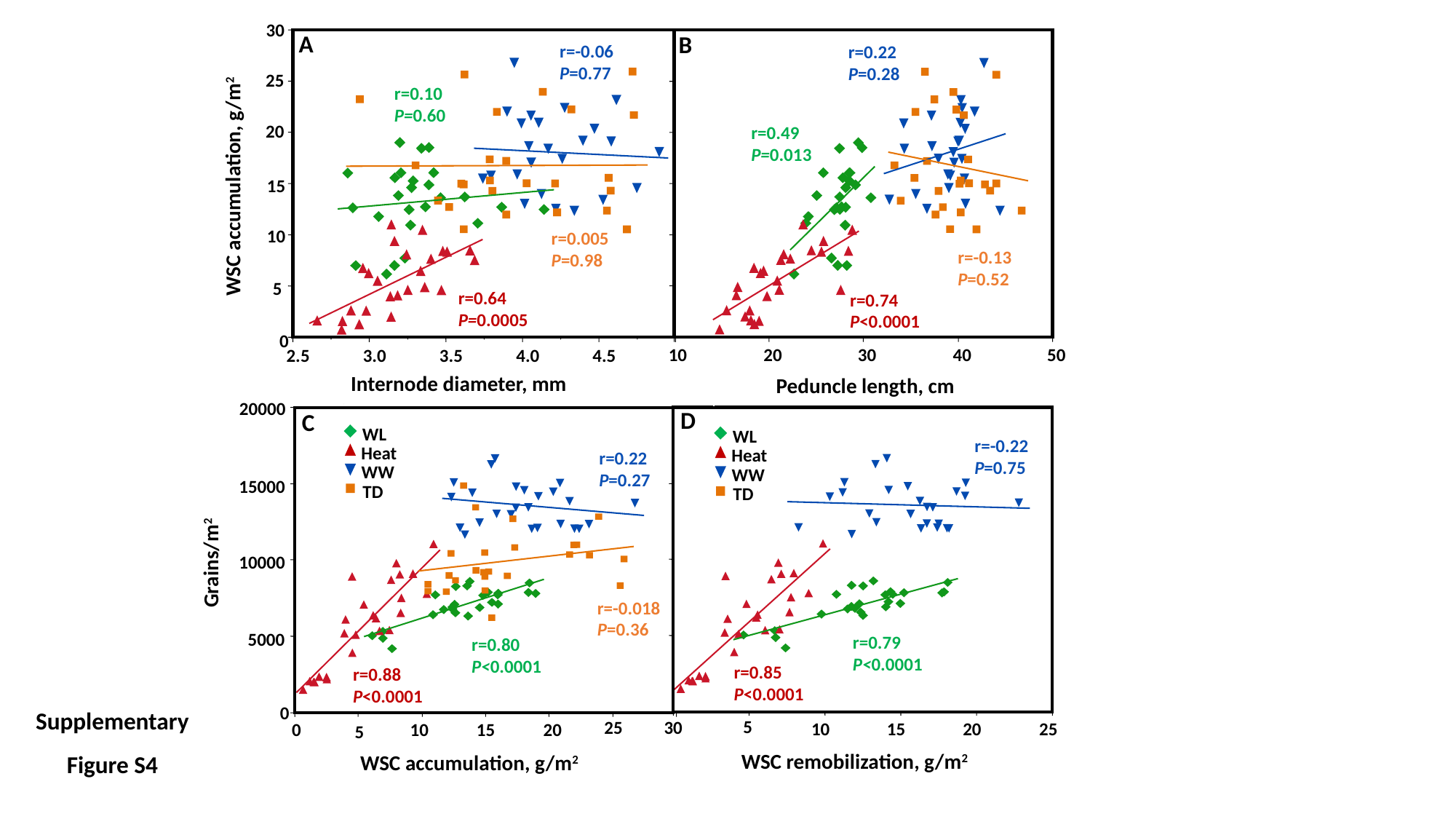

30
25
20
15
10
5
0
2.5
3.0
3.5
4.0
4.5
WSC accumulation, g/m2
r=-0.06
P=0.77
r=0.10
P=0.60
r=0.005
P=0.98
r=0.64
P=0.0005
Internode diameter, mm
A
B
10
20
30
40
50
r=0.22
P=0.28
r=0.49
P=0.013
r=-0.13
P=0.52
r=0.74
P<0.0001
Peduncle length, cm
20000
15000
10000
5000
0
30
25
0
10
20
15
5
Grains/m2
5
10
15
20
25
r=-0.22
P=0.75
r=0.22
P=0.27
r=-0.018
P=0.36
r=0.79
P<0.0001
r=0.80
P<0.0001
r=0.85
P<0.0001
r=0.88
P<0.0001
WSC remobilization, g/m2
WSC accumulation, g/m2
WL
Heat
WW
TD
D
C
WL
Heat
WW
TD
Supplementary Figure S4
